# Insights into product design students’ perception of, and engagement with, creativity in design education

**DOI:** 10.1007/s10798-022-09766-x

**Published:** 2022-11-09

**Authors:** Dermot McInerney

**Affiliations:** University of Limerick Faculty of Science and Engineering, Limerick, Ireland

**Keywords:** Creativity, Design education, Thematic analysis, Focus groups, Product design

## Abstract

Creativity is acknowledged as an essential component of the design process and considered a key design graduate attribute. Despite this assertion, several studies have highlighted that the teaching of creativity in design education is often implicit rather than explicit. Moreover, there is a paucity of research on product design students’ experience of creativity in design education, leaving a gap in knowledge relevant to how creativity may be better fostered in design education. In this study, reflexive thematic analysis was used to analyse the data from online focus groups conducted with product design students. The three themes constructed provide insights into how students perceive and engage with creativity. Theme 1, *the influence of social factors on creativity*, uncovers the diametric effect students’ social eco-system can have on their creativity. These include an aversion to being associated with it due to weight of expectation and negative perceptions around creativity, as well as the ‘invisible support system’ created by peers. Theme 2, *sanctuary seeking tendencies of novice design students*, reveals behaviours that oppose essential creative attributes: a freedom from risk and ambiguity and desire for certainty and achievability. Theme 3, *tension between passion for and pursuit of creativity*, outlines the conflict between participants’ ideologies and actions when pursuing creativity highlighting a reluctance to utilise structured creativity tools while gravitating towards unstructured methods such as ‘relaxed attention’. Together, the three themes form a picture of product design students’ complex relationship with creativity and importance of self-efficacy. The findings of this study make an important contribution to our understanding of design students’ perception of, and engagement with, creativity in design education. As such these findings are relevant for both design education and future creativity tool development.

## Introduction

The importance of creativity in society is indisputable (Ings, 2017) and since the emergence of *design thinking* in the 1990’s the phenomenon has become mainstream (Kelley & Kelley, 2013), with businesses recognising innovation, of which creativity is a central component, as essential to economic growth (Şener & Sarıdoğan, 2011). It is now recognised that everyone is capable of being creative (Runco, 2004; Sternberg, 2010) and that creativity can be cultivated within an educational context (Barak & Goffer, 2002; Karnia & Shalev, 2004). In fact, many researchers suggest that educational institutions have a responsibility to foster creativity in their students (Marquis & Vajoczki, 2012) and for many educational institutions creativity is a key graduate attribute (Osmani et al., 2015). Despite these assertions, there is little indication that deliberate creative thinking is being taught in design courses with several studies highlighting that the teaching of creativity in design education is often implicit rather than explicit (Christiaans & Venselaar, 2005; Oxman, 2004; Rodgers & Jones, 2017). Educational frameworks that enhance students’ creative thinking abilities do exist (Hargrove, 2012), but appear uncommon in practice as Wrigley & Straker’s (2017) study on design thinking in education across 51 courses in 28 international universities reveals. Moreover, there is a paucity of literature specifically about design students’ experience of creativity in design education, leaving a gap in knowledge relevant to how creativity may be better fostered in design education. In this study, focus groups were conducted with product design students about their experiences of creativity in design education, with the aim of developing our understanding of product design students’ perception of, and engagement with, creativity. Studying creativity in this field is pertinent as creativity is regarded as an integral component of the product design process (Cross, 1997) and due to its link with innovation, is an essential recruitment attribute for product designers (Yang et al., 2005), and a fundamental component of good product design (Rams, 1976). The three themes, constructed using reflexive thematic analysis, can help inform and shape future methods of creativity teaching in product design education. For the purposes of this paper the term ‘product design’ will be used to cover both product design and industrial design, as they refer to the same discipline and are interchangeable in education and industry.

### Conceptualisations around creativity

Rhodes’ analysis of creativity definitions led to the development of the first creativity framework known as the Four P’s (1961) in which creativity is presented as four strands: person (creator); process (creative process); press (environment); and products (creative outputs). Since then, several models of creativity have been proposed to conceptualize the phenomenon (Glăveanu et al., 2020), with many considering variants of the *person*, the *environment*, and the *field*. Glaveanu’s (2013) contemporary variation of these, the Five A’s Framework, adapts Rhodes’ (1961) Four P’s creativity framework and presents a socio-cultural view of creativity in which the individual cannot be abstracted from their context. By presenting creativity as five interrelated elements – actor (creator), action (creative process), artefact (creative output), audience (social environment), and affordance (material environment) – Glavenau aims to support this notion. For example, the actor can only exist in relation to an audience, while the action is influenced by the affordances embedded in the actor’s environment.

There is general agreement amongst creativity researchers about what defines creativity, with both novelty, and usefulness, deemed necessary (Finke, 1990; Teo & Waugh, 2010). Judgement of what is, and what is not creative, however, has proven much more challenging, and to date, there is no objective assessment tool for measuring creativity. Assessment of creativity typically relies on subjective judgement, such as the Consensual Assessment Technique (Amabile, 1982). There is also a distinction between a big “C” creative act in which a person brings something new and useful into a domain, and small “c” relating to everyday trivial creativity (Csikszentmihalyi, 1996). Big “C” can only be judged against the entire domain itself (such as the invention of the lightbulb), whereas small “c” can constitute what an individual considers new and useful based on their domain knowledge. Boden (1991) distinguishes between these as an idea that is original historically (H-Creative), and an idea that is original to the beholder (P-Creative). What most people perceive as creative would fall under P-creative or small “c” creativity, and it is this level of creativity that is discussed in this study.

### Domain specificity

The subject of domain specificity in creative theory – whether creativity is domain general or domain specific – is a contentious one (Baer & Kaufman, 2005). At the heart of this debate is whether components of creativity are transferable across domains. For example, is creative ability the same in different disciplines? Does creativity training transfer across domains? There is considerable evidence that supports the idea that creativity has both specific and general components (Plucker & Beghetto, 2004), and that there are common creative factors applicable across disciplines (Fasko, 2001; Marquis & Vajoczki, 2012). For example, common factors for fostering creativity include: supporting risk-taking (Kazerounian & Foley, 2007), increasing motivation (Torrance, 1972), building self-confidence (Cropley, 1992) or self-efficacy (Bourgeois-Bougrine et al., 2017), and developing a risk-free and supportive environment (Baer & Kaufman, 2005; Beghetto, 2010; Csikszentmihalyi, 1996). Also pertinent to this study, are the behaviours of creative novices. Several studies highlight common behaviours that cross all domains: a Personal Need for Structure (PNS) - a preference for well-ordered situations (Neuberg & Newsom, 1993); an aversion to ambiguity and confusion (Chirumbolo et al., 2004); and a Need for Closure (NFC) – an inclination to fixate on early ideas (Bourgeois-Bougrine et al., 2017; Van Hiel & Mervielde, 2003). Other noteworthy behaviours that inhibit creativity include: comparison to others (Kelley & Kelley, 2013); and social editing – modifying one’s behaviour to fit in (Ings, 2017).

While much can be learned from domain general literature, Glăveanu suggests we must ‘pay increased attention to the domain of the creation, the characteristics of the creator, and features of the situation’ (Glăveanu, 2013, p. 73). Hence, the intention of this study is to gain insights into the experiences of students within the domain of product design. To date, most of the research on creativity in design education focuses on the field of engineering, with few highlighting insights into the experiences or behaviours of product design students. Rodgers & Jones’ (2017) study highlights the importance of the wider socio-cultural system (including peers), in developing students’ creative skills, while also revealing students’ assumption that they are creative by virtue of their course of study (product design and architecture students). Cross’ (2004) comparison of expert and novice product designers reveal interesting dichotomies of practice, and offers insight into the challenge for design education. This study demonstrates that experts and novices differ in their problem solving strategies, while Silk et al.’s study (2021) indicates that novices (product design and engineering students) tend to solve design tasks as given.

### Creativity methods

Domain specific knowledge is acknowledged by most researchers as critical to creativity. Sweller et al. describe creativity as random generation based on specific domain knowledge, and propose that it can be enhanced by increasing one’s domain knowledge (2011). This reflects Csikszentmihalyi’s (1996) notion of the ‘prepared mind’, and the importance of knowing the domain first, before attempting to change it. Christiaans & Venselaar (2005) support this idea that in order to successfully solve problems, both domain specific knowledge and general process experience are necessary. The challenge for novice designers in being creative is that domain knowledge may initially be limited, and process experience takes time to develop. This also pertains to achieving what Csikszentmihalyi (1996) describes as a state of 'flow' in which optimal performance is achieved. Critical to achieving 'flow' is finding a balance of challenge and skill level where the challenge is neither undemanding nor stressful.

Multiple ideation tools and strategies exist as aids to problem-solving and innovation (Silk et al., 2021). The introduction of such tools to engineering courses has been shown to improve students’ creative outcomes (Bourgeois-Bougrine et al., 2017; Cropley & Cropley, 2010) and while this is not surprising, several researchers suggests that further research is required to establish the effectiveness of such tools (Haritaipan, 2019; Roy & Warren, 2019). Also, it is not clear how transferable creativity tools are across disciplines – Baer’s (1996) study on middle school students showed that creativity skills beneficial to poetry did not transfer to short stories. Furthermore, Bourgeois-Bougrine et al’s (2017) study highlights the importance of choosing an appropriate tool for the type of problem to be solved, the phase of the design process, and individual’s preference – something students’ are unlikely to master in one semester of instruction. Literature on creativity and design expertise typically focus on cognitive approaches rather than specific tool usage (Dorst & Cross, 2001; Lawson, 2006), highlighting commonly used un-structured creativity strategies such as: ‘relaxed attention’ (McKim, 1974); ‘mind wandering’ (Baird et al., 2012); and daydreaming (Kelley & Kelley, 2013).

## Method

### Design

The aim of this study is to gain insights into product design students’ experiences of creativity in design education. Data collected from three online focus groups (OFGs) conducted with product design students was analysed using reflexive TA. Reflexive TA was chosen for this study as it is well suited to analysing qualitative data captured from focus groups, where understanding about human experience, practice, and behaviour is sought (Braun et al., 2019). Focus groups were chosen as they encourage a range of responses which provide a greater understanding of the attitudes, behaviour, opinions or perceptions of participants on the research issues (Hennink, 2014) while also minimising moderator influence (Braun & Clarke, 2013).

Central to using reflexive TA is acknowledging the influence of the researcher on the outcome (Finlay, 2002) and noting ones position on the insider-outsider continuum (Holmes, 2020). In this study, the analysis of participant responses along with the construction of three themes are framed by my fifteen years of lecturing experience in product design. My undergraduate degree was in product design, and I continue to work in creative practice outside of my role in design education. The study took place in New Zealand during a sabbatical from my home university in Ireland. A critical realist ontological orientation was taken throughout. Central to this perspective is the belief there is not one ‘true’ reality, but only interpretations of reality (Terry et al., 2017).

A major challenge with the study was due to the COVID-19 pandemic and subsequent social restrictions imposed. As in-person focus groups were no longer possible, OFGs offered an alternate way to engage with participants who were already familiar with online learning. OFGs are reported to be consistent with the aims of traditional focus groups while having the additional benefit of offering greater control and equality for participants (Fox, 2017). In practice however, OFGs proved a challenge for moderating free-flow discussion due to the ‘turn-taking’ behaviour (to avoid speaker overlap), typical in online meetings.

### Participants and recruitment:

Participants were recruited from a three-year undergraduate programme in Industrial Design at Auckland University of Technology (AUT). To ensure a range of perspectives, participants included students from either end of the education cycle – 1st year and 3rd year (final year). At the time of the study, first year students had completed 1.5 semesters of the programme and third years 5.5. Twenty seven students expressed an interest and provided contact details for further communication, with twelve ultimately participating. This high attrition rate was a result of mid-semester disruptions caused by New Zealand’s second COVID-19 lockdown. The final number of participants was deemed appropriate to the research question, the method of analysis, and size of study (Braun & Clarke, 2013; Breen, 2006). The richness in this study is provided by deep and nuanced insights situated within the context of student’s personal experience, interpreted and represented in three novel themes. Saturation was not considered for determining sample size as it relies on “an understanding of meaning as transparent and obvious prior to analysis” and therefore not suitable for this method of analysis (Braun et al., 2019, p. 851).

Participant ages ranged from 18 to 22 with seven identifying as female and five as male. In terms of ethnicity, six identified as Pākehā (New Zealand European), five Asian and one undefined. As smaller group sizes are recommended for OFGs (Mann & Stewart, 2000), participants were split into three OFGs comprising four 3rd years in group one (Molly, Jane, Jack, & Toby), five 3rd years in group two (Helen, Sally, Ben, Judy, & Amy) and three 1st years in group three (Mary, Luke, & Tom).

The recruitment invitation stated that the focus group would discuss topics related to product design and not specifically on creativity. This indirect sampling strategy (Farvid, 2010) was necessary to ensure a variety of design students participated, and not just those who think of themselves as creative. This also mitigated against participants using pre-rehearsed definitions and ideas about creativity during the OFGs.

While the initial recruitment invitation was delivered in person, due to the COVID-19 pandemic, subsequent follow-up communications were conducted online. Potential participants received a participant information sheet and invitation to take part via email. Ethics approval was granted through AUT’s Ethics Committee prior to participant recruitment. Conflicts of interest and power imbalances were minimised as the researcher was not involved in teaching with, or assessment of, any participants at the time of the research.

### Data collection

Three categories of prompts were developed (totalling twelve prompts) to stimulate open discussions on students’ personal experiences of creativity, within their design education (see Table 1). This semi-structured approach offered an opportunity for participants to express their thoughts and feelings on themes related to the research question. For this study, it was deemed important to situate students’ experience of creativity within a wider interconnected context, as informed by Glaveanu’s Five A’s creativity framework (2013), therefore categories were devised to capture a wide range of experiences with creativity. The Category 1: *Understanding and perception*, was included to frame students’ experience of creativity in relation to their knowledge and perception of design and creativity. Category 2: *Engagement in practice*: sought to generate discussion around their actions during engagement with creativity. Category 3: *Its place in design education*: aimed to understand their experience of creativity in design education. The prompts were first trialled during a pilot study and then revised for use in this study. Each OFG took approximately 90 min and was conducted using Microsoft Teams. Each session was moderated by the researcher. Audio was recorded and then transcribed verbatim, student data anonymised, and pseudonyms assigned. In line with ethics approval, all audio recordings were subsequently destroyed.

**Table 1:** Focus Group Prompts

Category:	No.	Prompt:
Understanding & perception of creativity	1.	(a) What do you think makes a good design? (b) Can you give an example of a ‘good design’ product you own or have used in real life, and the attributes that make it so?
	2.	(a) What is your perspective on creativity and (b) what does it mean in your life?
	3.	How do you know when a product is creative?
Engagement with creativity in practice	4.	Do you think you are a creative person? Why, or why not?
	5.	Tell me about creativity in your design process.
	6.	Can you judge or assess the creativity of your work?
	7.	How do you get into a creative head-space?
	8.	Do you practice or nurture being creative? How so?
	9.	Are there any things that hinder your creativity?
Creativity in design education	10.	Is there an expectation for you to be creative as a design student?
	11.	Is creativity discussed with your tutors or peers?
	12.	How has your creative ability improved throughout your design education?

### Data analysis

Data analysis followed Braun and Clarke’s (2006) six-phase guidelines for reflexive TA to ensure consistency of approach throughout the procedure (see Table 2). Reflexive TA is particularly suitable for exploratory research as it allows unexpected findings to emerge. Rather than using pre-made codes to test a hypothesis, reflexive TA uses an inductive approach to generate codes from the data out of which themes are constructed (Braun et al., 2019). It is also important to note that reflexive TA is a distinct and independent qualitative method and should not be confused with its TA counterparts – a *codebook* approach or a *coding reliability* approach. Reflexive TA sits at the interpretive end of the descriptive-interpretive continuum and operates solely as a qualitative technique within a qualitative paradigm – a ‘Big Q’ approach (Terry et al., 2017).

**Table 2:** Braun and Clarkes six-phase guidelines for reflexive TA

1.	Familiarising yourself with the data and identifying items of potential interest
2.	Generating initial codes
3.	Constructing prototype themes
4.	Reviewing potential themes
5.	Defining and naming themes
6.	Producing the report

Following *familiarising yourself with the data*, step one of Braun and Clarke’s six-phase guidelines, initial codes were succinctly and systematically applied to the data (step 2). Codes are used to identify the most basic segment of raw data, and captures what is interesting about it (Boyatzis, 1998). A total of 604 semantic (descriptive) and latent (interpretative) codes were generated from the dataset. A sample of these codes along with their respective raw data (participant responses) are presented in Table 3. For readability purposes participant data was cleaned up, while ensuring not to change the meaning of data.

**Table 3:** Samples of codes generated from the data

*Raw data*	*Code*
*“Creativity… is one of those things that makes me feel a bit anxious…, because it’s like if people say like someone’s creative, it sets a bar or expectation where you have to do certain things.”* (Toby)	Undermining of self-efficacy
*“I think you shouldn’t have fear of failure. It’s a pretty safe environment within University. At the end of the day, the worst that can happen is that you get a bad grade - which is not the end of the world.”* (Luke)	‘Safe’ environment facilitates creative freedom
*“When I was doing it, I was definitely nervous, because I didn’t quite know what I was doing [with the design] and it left me feeling like… it wasn’t the typical way to do something.”* (Molly)	Anxious due to the unknown/uncertainty
*“I think it’s that drive [to be creative] that you were talking about. It’s almost addicting, I want to say addicting, but maybe that’s a strong word. It keeps you going.”* (Jack)	Excitement of being creative is motivating
*“Like I do it [use creativity tools], but I don’t think good ideas come out by doing it. It feels more like torture sometimes.”* (Judy)	Ideation methods can be a chore
*“Hugely important and its super nice to be around people that think in the same way as you and it can give valuable critique to your work, and value, and insight. It’s really, really important.”* (Luke)	Engagement with people is vital
*[On how to be creative] “I like to lie flat, anywhere, on a floor on a couch, in a like mummified position, close my eyes, try not to sleep and try and get into that headspace. Sometimes I dance around as well.”* (Jane)	Creative through relaxed attention
*“Once I came up with that [my idea], it was like, oh I don’t feel like coming up with any other ideas, … It was still like, no-one else in the class had the same thing as me, so I was like, cool, I can claim that one, that’s going to be my one! (Happily excited!)”* (Mary)	Importance of design novelty

On completion of step 2, *generating initial codes*, twenty three sub-themes were created by organising codes into shared meaning-based patterns related to the research question (Braun & Clarke, 2006). Creating sub-themes was a useful next step and made the construction of prototype (candidate) themes easier (step 3). Seven prototype themes were then reviewed by collating related data along with a draft summary for each prototype theme (step 4). This facilitated the verification of prototype theme’s quality and boundaries, and ensured the associated raw data accurately portrayed shared meaning across the entire dataset. Following much iteration and revision of the prototype themes, three final themes were constructed (see Table 4). These were then named and fully defined to accurately reflect each theme (step 5) and are presented in the *Analysis* section of this paper.

**Table 4:** Sub-themes, candidate themes and final themes

Initial sub-themes (23)	Prototype themes (7)	Final themes (3)
Reliance on unstructured approaches Dislike towards unnatural methods Peers as a creativity method Use of “gut” (intuition) in decision making Consequences of comparison Need for novelty Social expectation Internal expectation & pressure The creative label Cultural expectations The joy of being creative Creative confidence Supporting uncertainty Freedom from risk Explicit vs. implicit focus on creativity Challenges to creativity Personal learning experience Constraints of working space Judgement of creativity Social support Achievability Dislike of ambiguity Desire to be creative	1. Reliance on unstructured approaches	1. The influence of social factors on creativity
	2. Tension between passion for and pursuit of creativity	2. Sanctuary seeking tendencies of novice design students
	3. The joy of being creative	3. Tension between passion for and pursuit of creativity
	4. Creativity seldom occurs in a vacuum	
	5. Personal preferences for how to be creative	
	6. Sanctuary seeking behaviour	
	7. The importance of creative confidence	

## Analysis

Three themes were constructed from the data generated from OFG discussions: *(1) the influence of social factors on creativity*, *(2) sanctuary seeking tendencies of novice design students*, and *(3) tension between passion for and pursuit of creativity.* The themes present patterns of shared meaning across all OFGs, and provide insights into students’ perception of, and engagement with, creativity.

### THEME 1: the influence of social factors on creativity

*The influence of social factors on creativity* captures a range of opposing experiences triggered by students’ socio-cultural eco-systems. Participant responses suggest that these can have both positive and negative influences on students. For example: Molly’s comment “people just assume you are super creative because you do design”, is a sentiment shared by several participants and highlights the weight of expectation associated with the creative label. Rather than being worn as a badge of honour, several participant comments suggest it is an unwanted imposition:

“If I try to show some of what I’ve done to my friends, I do feel like it has to be creative, to validate that I’m taking the course.” (Mary)

Mary’s comment exposes a latent requirement for students to match society’s view (label) of creativity, even at the fledgling stage of becoming a designer. It also presents a scenario where society (in this case Mary’s friends, who are not designers) is perceived as judgemental, and to whom design students feel obliged to prove their position. Even within a nurturing and congenial educational environment, students are not shielded from the pressure of expectation. In fact, it can be amplified: “Everyone here is really creative, it’s expected from the [design] school” (Helen). Helen’s comment presents a predicament for design students – if you are in the school then you are, or at least should be, creative. It also assumes that the creative label is already in place prior to enrolment, and not necessarily due to the programme curriculum. A sensitivity to these external pressures is apparent throughout the OFGs, even when it is not clear what the bar of expectation is:

“Creativity makes me feel a bit anxious, because if people say someone’s creative, it sets a bar or expectation where you have to do certain things.” (Toby)

Toby’s comment highlights a lack of confidence with his status as a ‘creative’ and an uncertainty about his ability to fulfil expectations. A certain level of frustration at this fixed identity is also obvious, as students’ confidence naturally fluctuates from high to low. Sally’s comment that the creative label “can give you more confidence, but also add more pressure” underlines the idea that students’ self-efficacy (Bandura, 1989) is not static and can be influenced by external social factors. This comment also highlights how social pressures were polarising, with some participants feeling both empowered and pressurised by it, while others were indifferent. Although “certain things” (what constitutes being creative) was not defined during the OFGs, discussions on what creativity is not associated with, were insightful. In many cases participants’ aversion to the ‘label’ was ascribable to the view that creativity cannot co-exist with academic ability. Amy’s comment presents a specific conception of creatives within some sections of society:

“I thought about not wanting to be labelled as creative, and the social stereotypes and concepts that society creates around creativity. If you’re creative, it seems to be perceived as less academic, or you know you are part of this type of [non-academic] category.” (Amy)

The conception that one cannot be both academic and creative is a myth some students have been subjected to. Here, Amy demonstrates an unwillingness to be defined by such a limited perception of the phenomenon. A break-away discussion in the third OFG reiterated this perspective, attributing it to certain cultural backgrounds. One cohort of participants openly talked about the negative perception of creative pursuits that exists within their cultural backgrounds:

“So, if it were my friends and peers, creativity would be seen as a good thing, but within my family and culture is not seen as a good thing in comparison to being academic. Creativity equals failure”. (Judy)

Here again we see the misconception that creativity and academic ability are mutually exclusive. In earlier comments the creative label was mostly presented as something that held prestige, therefore participants still pursued it despite any anxiety caused by it. In this scenario, the creative label only holds negative connotations, and is something participants wished to avoid.

In contrast to the potential negative influences of the creative label, is the invaluable support that social factors provide. While only some participants reported experiencing pressure due to social expectation, all participants highlighted the support social interactions provide. For example:

“He [the design tutor] was really awesome about it and [encouraged me] to persist with it and I got a really cool outcome. If I didn’t have that, other people’s comments and an uncertainty because it was quite unknown for me, I would have tapped out [stopped].” (Molly)

Molly’s lack of experience and confidence contributes to her insecurity – in this case, the challenge of her design project and her skill level were not in 'flow' (Csikszentmihalyi, 1996). This comment highlights the nurturing affect social factors can have on novice designers. Without the support of her tutor, the ensuing anxiety may have made her quit. Several participants also expressed the positive influence of social factors on their design work: Jack spoke about project clients who gave “assurance”; Toby stated how positive feedback from product users provided “validation”; and Jane mentioned ever-present family members who provided “support”. These comments show how positive interventions can act as a counterbalance to uncertainty and self-doubt.

The most frequently stated social factor across all OFGs was that of a reliance on peers. Amy’s comment: “I think the time I am most productive is after speaking with my peers and being surrounded by others” highlights the positive effects of this ecosystem. Described by Ben as “an invisible support system”, discussions around it portray it as predominantly informal and self-regulating. Participants sought peers at different times of the design process and for different purposes. For example, as a “fact checker” (Tom), or to “bounce ideas off” (Mary). Other participant comments demonstrate a reliance on peers for feedback and support, or to validate creative success.

### THEME 2: sanctuary seeking tendencies of novice design students

The theme *sanctuary seeking tendencies of novice design students* identifies participants’ desire for a cognitive safe-haven: a freedom from risk and ambiguity, and desire for certainty and achievability. Participants’ responses across the dataset present several factors that support this theme. A desire for a risk-free working environment was widely reported by participants, with many feeling at their most creative once a fear of failure and pressure of expectation were removed. Participants such as Molly were enthused and liberated when afforded this freedom: “I definitely thrive in a situation where you can’t get it wrong, and you just get to play” (Molly). In this scenario it is the freedom from consequence that forms a sanctuary around students in which full creative expression is afforded. In other scenarios, sanctuary relates to achievability – a certainty that participants’ design goals were possible. For example:

“I pretty much stuck with the one idea that I liked. Once I came up with that it was like, oh I don’t feel like coming up with any other ideas, because I know that’s the one I’m going to stick to. I knew that I could laser cut it out easy. It was materials that I knew that I had.” (Mary)

Mary’s comment reveals how relief and assurance were attained once she was certain of fulfilling the project objectives and had a clear and achievable design direction. While Mary also seeks a “you can’t get it wrong” situation, her need for certainty is at odds with being creative as it results in NFC behaviour (Van Hiel & Mervielde, 2003). In this scenario, Mary’s desire for sanctuary inhibits opportunities for creativity. This desire for certainty is understandable, as uncertainty during participants’ design process led to frustration, annoyance, and an attenuated willingness to pursue ideas. For example:

“I know most of the people have creativity, but I’m not sure is if that is useful or is that enough. We get a lot of ideas in our life, but when we are in a project, I don’t know if that works. That’s the biggest question for me, I have a lot of thoughts, but some of them are not working and some of them are bad, but I don’t know how to judge it.” (Sally)

Sally’s comment reveals a conundrum for students that have ideas but are unable to realise these. Her lack of domain knowledge leaves her at an impasse, unable to tell whether her ideas are feasible or have any merit. The uncertainty and ambiguity of Sally’s predicament leaves her discouraged and undermines her self-efficacy.

Contrasting preferences for types of project briefs was another indicator of students’ desire for sanctuary, with some students preferring constrained briefs, whilst others preferred open briefs. Those who preferred open briefs liked that they could pursue their passion and values, while others felt that open briefs were “too overwhelming” (Judy) due to the endless possibilities and lack of focus. Each participant had their ‘comfort zone’ as Amy’s comment highlights:

“I think when you have your own choice [of project brief], the way that you choose to express your creativity is really beneficial and it can be easier, because you can choose how you want to outlet it. The cool thing is you have freedom.” (Amy)

In this example Amy describes her ideal project scenario. The freedom afforded by a self-selected brief compliments Amy’s creative tendencies and desire for unhindered expression. For Amy, this is what matches her ideal scenario with uncertainty of achievability not an issue. In contrast to this example, other participants comments suggest a dislike for open design briefs due to the ambiguity of the starting point. Toby provided an interesting analogy: “it’s like having a puzzle and having to assemble it, rather than having to go to the store and finding the right puzzle.” For participants like Toby, his ‘comfort zone’ was afforded by well-defined design briefs that provided certainty in their starting points.

A further challenge to participants’ preferred state of sanctuary emerges from a need for novelty – a desire to create something unique in their design projects. Deemed as desirable by participants, achieving this goal resulted in delight. However, the challenge of producing designs that differed from those of classmates caused much anxiety and uncertainty until novelty was achieved:

“Sometimes doing it [a design project] with the whole class you’ve got the exact same thing to do, it can be stressful thinking you’re going to come up with the same thing as someone else and you feel less creative as you feel like someone has done it.” (Molly)

Molly’s comment shows how the need to be unique can become a self-imposed design objective for some students. It presents a scenario where duplication must be avoided as comparison may come at a cost. In most cases participants stated that comparison was inevitable, but in general they tried to avoid it. Often, it was the need for assurance that led participants to compare their work with online examples or the work of classmates. Conversely, this often had the opposite effect with participants reporting that comparison undermined confidence and “can be stunting” (Luke) to their creative process – This also reflects Molly’s desire for uniqueness. Although participants mostly agreed that comparison was not a good idea, it was difficult for students not to compare to work online or with that of classmates:

“You can’t really compare, but with so much media out there, it’s pretty hard not to, even though you may not be comparing yourself on a scale that is valid at all.” (Jane)

Jane’s comment highlights that students are aware it can be a senseless act. As with many of the participants’ responses, contrasting views were also voiced. While the need for novelty can lead to anxiety and undermining of confidence, one participant did state that comparison led to “healthy competition” within the class (Ben). This alternate opinion provides evidence that participant experiences are dependent on the individual’s perspective.

### THEME 3: tension between passion for and pursuit of creativity

The final theme, *tension between passion for and pursuit of creativity*, outlines the conflict between participants’ aspiration to be creative and actions to realise this. Responses from the opening category of OFG prompts *understanding & perception of creativity* offered interesting insights into participants’ perceptions of creativity and its place in the design process. All acknowledged the importance of creativity in design and demonstrated reasonable understanding of its place in the design process. Also noteworthy from this initial discussion was participants desire to be creative. For example: “It’s fulfilling, it’s also what keeps me motivated. I need to be creative in some way at all times. I think this is what keeps me moving.” (Amy). In this comment, we see how creativity is much more than just an appealing attribute for Amy but is essential in her life. This need to be creative is also echoed by other participants through animated descriptions such as “it’s addicting” (Jack) and “almost like an adrenaline rush” (Toby). The use of such terms highlights the excitement and joy associated with the creative act. For Amy, the pursuit of creativity was not just limited to the domain of design but carried across other daily activities - “You can have different outlets for creativity and for me its food, it’s creating recipes, it’s constructing, using different components, and constructing a meal”. It is clear from this scenario that Amy’s creative self-image is independent of design and her status as a design student.

Discussions around enabling creativity revealed a variety of disparate methods used by participants to facilitate being creative, the most common of which was that of engaging with peers. Tom’s excerpt summarises many of the benefits of peers in facilitating creativity:

“What’s so unique about class is having creative people around, other people who understand your ideas. When sitting in class I’ll bounce an idea off, and I’m not even worried that someone will take my idea, and they’ll be like oh that’s pretty cool. And I’ll talk to them about it and that helps me understand what I’m trying to do as well.” (Tom)

Tom’s comment reveals the casual but supportive nature of peers as a creativity method. In this scenario participants’ education takes place in the design studio environment which provides a permanent workspace in which peers can engage. This comment reveals that the level of investment required is flexible and can accommodate simply “bouncing an idea”, or having a protracted discussion on design.

In contrast to participants regular use of peers as a method to facilitate creativity, few participants declared engaging with structured creativity tools, despite many being familiar with tools such as Brainstorming and Lotus Blossom. In some instances, participants only engaged with such tools to fulfil assessment criteria:

“Yeah, it’s not natural for me, but I mean I do it [try creativity tools]. I do Mmind-mapping, and Bbrainstorming and stuff. That’s kind of to check the boxes for the process document [a document used for assessment purposes]. Yes, I might get a good idea out of it, but I don’t feel this is the best way for me.” (Judy)

Follow up comments reveal the rationale for Judy’s apparent indifference to creativity tools. Both Mary and Judy present a strong dislike towards the un-natural feel of prescribed tools, citing that they “forced” ideas (Judy), and using them felt like “such a chore” (Mary). The overall sentiment of the group was that use of creativity tools often resulted in few new ideas. Furthermore, they required significant effort for insufficient reward. Other participants stated that regardless of their attempts at using tools, if they were not in a creative mindset, their effort was often futile. Several participants reported trying methods without success. Jane’s excerpt highlights this common scenario:

“I have a lotus [Lotus Blossom tool] right above me. It’s not a very successful one. I attempted it yesterday, it did not make me very creative. I did not lotus! I don’t think I was in the right head-space. I think I just tried a whole bunch of different methods over the past week, but not feeling particularly inspired.” (Jane)

Jane’s comment highlights the frustration of struggling through creativity methods with little success. This conundrum is not unique to Jane, and attaining this “right head-space” was often the first step for students in being creative. Several participants reported using diverse activities with the aim of attaining a creative mindset, in favour of finding and using structured creativity tools. Participant responses include a wide range of diverse activities such as: “getting hyped” or “having a dance” (Molly); “listening to really loud music” (Toby); “lying on the floor in a mummified position” (Jane); “playing video games” (Sally); “having a long shower” (Jack); and “doing unrelated tasks” (Judy). Some of these activities match those reported in Csikszentmihalyi’s (1996) study of creative people’s pursuit of 'relaxed attention' (McKim, 1974) giving credence to participants’ curious behaviour. However, participants’ responses clearly indicate that this method does not guarantee success and it is typically an aspirational approach – “hopefully something comes from there” (Helen).

## Discussion

The aim of this paper was to gather insights into undergraduate product design students’ perception of, engagement with, creativity. Data collected from OFGs was analysed using reflexive TA from which three themes were constructed; (1) *the influence of social factors on creativity*; (2) *sanctuary seeking tendencies of novice design students*; and (3) *tension between passion for and pursuit of creativity*. Discussions on each theme along with an overall discussion on the interrelated nature of the findings are presented below.

### Theme 1 – the influence of social factors on creativity

*The influence of social factors on creativity* highlights both positive and negative influences of the wider socio-cultural factors on students’ perception of and engagement with creativity. It presents multiple social factors that form an eco-system of support but also expectation around students. The notion that creativity does not happen in a vacuum is not new. Rhodes (1961) highlighted this in his development of the 4P’s^1^ creativity framework in which he uses the term 'Press' to describe environmental influences that help condition and inspire creative outputs. However, in Rhodes’ framework, the associated social factors relate more to the creative action and output of individuals, and less to intangible social influences that impact individuals’ engagement with creativity, as highlighted in this theme. The importance of a supportive environment in fostering creativity is also well-established (Baer & Kaufman, 2005; Beghetto, 2010), though the negative experiences reported by some participants in this study highlight social factors that oppose this ‘congenial environment’ (Csikszentmihalyi, 1996). Participants displayed a sensitivity to the creative label and an aversion to being associated with it, due to the weight of expectation it carries, and its perception as non-academic in some cultural backgrounds. This presents a more complex view of students’ relationship with creativity than that of Rodgers and Jones’ (2017) study in which design students believed that they are creative due to their programme of study, and Sawyer’s (2006) assertion that people typically use ‘creativity’ as a complimentary term of praise.

The positive influence of social factors is also recognised in this theme with participant responses verifying the importance of the wider socio-cultural system (Rodgers & Jones, 2017) and the desire for creative people to surround themselves with people who support their work (Harrington, 2011). While multiple sources of social support were highlighted, the 'invisible support system’ created by peers was deemed the most significant, offering reassurance, feedback, support, and validation of creative success.

While the social factors presented in this theme pervade all participant lives, participants describe differing levels of awareness and influence of each. This variation of participant experience reflects Murray’s (1938) notion of 'beta press', where reality is related to an individual’s interpretation of the situation. The pressure and anxiety experienced by participants as a consequence of a variety of socio-cultural factors is noteworthy, as little is published on these experiences or how they might be mitigated against in the design studio. Further investigation into how these positive social factors can be supported while simultaneously mitigating against factors that oppose the congenial environment would be worthwhile, particularly in an online education setting.

### Theme 2 – sanctuary seeking tendencies of novice design students

Theme two, *sanctuary seeking tendencies of novice design students*, paints a picture of students’ preferred cognitive conditions when engaging with creativity in a design education environment. Participant tendencies reveal a desire for certainty and achievability, and freedom from risk and ambiguity. It is noteworthy that these are contrary to essential creative attributes, such as a tendency towards risk-taking (Kim, 2020) and an ability to work in ambiguous contexts (Kazerounian & Foley, 2007). While professional designers accept uncertainty and ambiguity as part of the design process, intentionally leaving the process open and ambiguous at times (Lawson, 2006), participants of this study report struggling with this approach. Cross (2004) suggests that experts develop an ability to work in this fashion through years of experience, therefore it should be expected that novice designers will lack this capability and default to working within their comfort zones.

Multiple participant responses suggest that a lack of technical understanding contributed to uncertainty about the viability of their designs. The reasons for this frustration and anxiety are clear: without both process and domain knowledge the ability to solve problems successfully is compromised (Christiaans & Venselaar, 2005). This also aligns with Csikszentmihalyi’s (1996) Flow Theory, where 'flow' is achieved when there is a balance between challenge and perceived skills. However, when the challenge outweighs the perceived skills, performance anxiety ensues (Fullagar et al., 2013). This supports participants desire for a 'you can’t get it wrong’ setting, in which the design challenge is undoubtedly achievable. Frustration and anxiety can be averted by the freedom from consequence afforded by this scenario. It also verifies participants personal preferences for design project type (open or closed design briefs), that match their comfort zones. Also aligned to students’ desire for achievability, is their need to create solutions that differ from the work other designers, including their classmates. Multiple responses suggest that design novelty is often deemed by students as a measure of project success. As a result students can focus excessively on comparison which has the potential to undermine participant’s self-efficacy (Bandura, 1989).

The sanctuary seeking behaviour of students highlighted in this theme raises several considerations for how creativity is fostered in design education. The challenge for design tutors is how to subvert any undesirable tendencies that oppose essential creative attributes. Bourgeois-Bougrine et al.’s (2017) study demonstrates how a NFC behaviour can be subverted; while other essential creative attributes, such as risk-taking and an ability to work in ambiguous situations, can be encouraged with a supportive and risk-free environment (Cropley & Cropley, 2010; Rodgers & Jones, 2017). Additional challenges highlighted by students’ sanctuary seeking behaviour are a consequence of an imbalance of design task, and student skill (Flow Theory). The challenge for design education is in balancing these variables across a cohort ranging in creative ability. Future studies should investigate how student skill and design challenge can be individualised in a design education setting.

### Theme 3 – tension between passion for and pursuit of creativity

The final theme, *tension between passion for and pursuit of creativity*, outlines patterns of activity across the dataset that raise interesting questions about students’ desire to be creative and their actions to achieve this. Discussions initiated by the first category of prompts helped frame participants’ understanding of creativity and its place in the design process. From these, it is clear that participants value creativity while also demonstrating good awareness of its necessity within the design process. Furthermore, motivation and desire to be creative are also apparent. Intrinsic motivation, a prerequisite for being creative (Amabile, 1982), along with the joy of being creative (Csikszentmihalyi, 1996) was evident from participants’ passionate responses.

However, despite participants’ acknowledgement of the importance of creativity in design, along with expressing an explicit desire to be creative, there is discord between their aspiration to be creative and their practical pursuit of creativity. Although participants acknowledged being familiar with creativity tools, few reported these as useful and instead relied on less structured approaches to ideation. These include talking with peers; and 'relaxed attention' (McKim, 1974) – the pursuit of unrelated tasks to facilitate the emergence of ideas. This raises the question: with so many creativity tools in existence (Roy & Warren, 2019), why do participants demonstrate a reluctance to utilise structured creativity tools but gravitate towards unstructured methods?

Participants’ responses offer some insights into this behaviour – structured creativity tools can be perceived as a ‘chore’, or unsuited to an individual’s method of working – reflecting individuals’ cognitive styles & preference for strategies and tools (Silk et al., 2021). Alternative reasons for students’ avoidance of structured creativity tools may be due to the correlation between poor mastery and weak results (Bourgeois-Bougrine et al., 2017). It is probable that participants have not spent sufficient time learning how to use these tools, resulting in ineffective outcomes and reluctance to try again. Furthermore, creativity tools are typically suited to certain types of design problem and parts of the design process (Roy & Warren, 2019), therefore inappropriate use will also result in poor outcomes, reinforcing the tool as an ineffective ‘chore’.

In contrast to this, it is easy to understand participants’ tendency towards unstructured methods such as talking with peers and 'relaxed attention'. Unlike many creativity tools, peers can adapt to multiple problem spaces and also offer a spontaneous ‘soundboard’ for generating ideas. Furthermore, the reactions of others to students’ creative work can be an important ingredient in developing their creative skills (Daly et al., 2016). Likewise, 'relaxed attention' offers flexibility of use, and is commonly used in problem-solving activities and creative endeavours (Csikszentmihalyi, 1996). This behaviour also correlates with Sweller et al’s (2011) random generator concept of creativity as it frees up the subconscious mind in which random generation can occur. In addition, both methods are easy for students to engage in. Interestingly, participant responses suggest that 'relaxed attention' gives inconsistent results, with most pursuing it in the hope of ideas emerging. Despite this, the method is still widely used by participants.

The tension highlighted in this theme, between students’ desire to be creative and their ability to actualise creativity, has implications for creativity tools and strategies in design education. A novice-centred approach in which students’ cognitive styles & preferences are considered would benefit both the development of future creativity tools and methods. While this theme has provided new insights into students’ partiality to unstructured creativity tools and avoidance of structured tools, additional research specifically on this topic would be of benefit to how students’ creativity is developed in design education.

### Relationship of themes

Consideration of creativity from the perspective of product design students has provided several novel insights that begin to form a picture of their experiences with creativity. Together, the three themes presented in this study highlight a range of multifaceted and complex factors that influence students’ perception of, and engagement with, creativity. In this way, the study’s findings reflect Gláveanu’s (2013) Five A’s framework in which the complexity and relational nature of creativity is emphasised. The findings of this study highlight the interrelationship of ‘actor’ (student), ‘action’ and ‘audience’, and how this forms the creative experiences of product design students. The importance of the ‘actor’s’ self-efficacy – the belief in their capacity to succeed in a particular situation (Bandura, 1989), is also prevalent across themes, and highly dependent on the student’s experiences of creativity during their design education.

### Limitations

While the themes presented in this study provide useful insights that inform our understanding of product design students’ perception of, and engagement with, creativity, the findings are limited by the small sample size and inclusion of participants from a single product design programme. There is an opportunity for further research to include participants from a diversity of design institutions and cultures in order to extend these findings. For a more nuanced understanding of how design education affects students’ creativity, a comparison of first year and final year students’ experiences would also be worthwhile. Furthermore, further research would be required to establish whether the findings of this study are transferable to other domains, or are domain specific.

## Conclusions

The findings of this study – captured in three themes – provide novel insights into product design students’ perception of, and engagement with, creativity. The study makes an important contribution to our understanding of design students’ experiences with creativity in a subject area of limited literature and highlights several significant novice tendencies that educators should be aware of.

The first theme; *the influence of social factors on creativity*, highlights the sphere of influence created by a combination of multiple social factors that can have both a positive & negative impact on design students’ perception of, and engagement with, creativity. These include an aversion to being associated with it due to weight of expectation and negative perceptions around creativity, as well as the ‘invisible support system’ created by peers. The theme draws attention to the challenge of how to support positive social factors, while mitigating against negative social factors, in a design studio environment.

The second theme, *sanctuary seeking tendencies of novice design students*, presents students’ preferred cognitive conditions when engaging with creativity: a freedom from risk and ambiguity, and desire for certainty and achievability. An imbalance of design task and student skill is a key influencer of this behaviour. Design educators should consider how these tendencies – that oppose essential creative attributes – might be subverted in the education cycle.

The final theme, *tension between passion for, and pursuit of, creativity*, outlines the conflict between participants’ ideologies and actions when pursuing creativity, highlighted by a dislike of structured creativity tools and preference for unstructured creativity methods such as ‘relaxed attention’. The theme draws attention to a need for a novice-centred approach in the development of future creativity tools and methods, while also emphasising the importance of individuals’ cognitive styles & preference in the application of creativity tools and strategies in design education.

The themes as a whole highlight the complexity and interrelatedness of ‘actor’ (student), ‘action’ and ‘audience’, and helps to build a holistic picture of creativity from the product design students’ perspective. Furthermore, the importance of the ‘actor’s’ self-efficacy is seen across themes and is highly dependent on the student’s experiences of creativity during their design education. The findings provide insights that should be of benefit to those fostering creativity in a design education setting, while also highlighting additional topics for further research.

## Footnotes

1 The 4P’s of Rhodes 1961 framework refer to the four different strands of creativity: *Person, Process, Press*, and *Product*.
